# Chloroform in Chorea

**Published:** 1848-05

**Authors:** 


*Chloroform in Chorea.*—In the Medical Gazette of February 4th, it is stated that Mr. Sibson of Nottingham, has been using the chloroform in chorea, and that "the movements during the anæsthetic state" were suspended. Without in any way anticipating what may be Mr. Sibson's opinion as to the real value of this agent in chorea, I beg leave to state that I have myself used it in this protean disease, as far back as December 18th, and with a result which has not encouraged me to try it again, I exhibited it three times, and certainly it effectually checked the violent convulsive motions, when the patient continued in her quasi trance, on recovering from which, however, the grotesque movements peculiar to chorea were as bad as ever. The child expressed herself as pleased, and refreshed with the short sleep she experienced under the influence of the remedy, and did not in any way suffer from it.

